# Operating during pregnancy: A needs assessment among surgical residents in Austria

**DOI:** 10.1016/j.heliyon.2023.e15863

**Published:** 2023-04-29

**Authors:** Nadja Taumberger, Philipp Foessleitner, Petra Pateisky, Bettina Toth, Taja Bracic, Karin Windsperger

**Affiliations:** a Department of Obstetrics & Gynecology, Medical University of Graz, Graz, Austria; b Department of Obstetrics and Gynecology, Division of Obstetrics and Feto-maternal Medicine, Medical University of Vienna, Vienna, Austria; c Medical University Innsbruck, Department of Gynaecological Endocrinology and Reproductive Medicine, Innsbruck, Austria

**Keywords:** Female physicians, Operating during pregnancy, Maternity protection law, Equal rights

## Abstract

In Austria, female physicians must immediately disrupt their surgical training as soon as their pregnancy is announced. In Germany, surveys on the topic of “female surgeons performing surgery during pregnancy” led to a reform of the German Maternity Protection Act, which came into force on January 1, 2018, and allows female physicians to perform risk-adapted surgery during pregnancy at their own request. However, in Austria, such reform is still pending. The study aimed i) to assess the current situation of how pregnant female surgeons handle their training under the actual restrictive legislature in Austria, especially in context of operative activity, and ii) to identify needs for improvements. Therefore, a nation-wide online survey, initiated by the Austrian Society for Gynecology and Obstetrics and the Young Forum of the Austrian Society of Gynecology and Obstetrics, was performed from June 1 to December 24, 2021, among employed physicians working in surgical specialties. To conduct a general needs assessment, the questionnaire was made available to both female and male physicians in all positions. In total, 503 physicians participated in the survey, of which 70.4% (n = 354) were women and 29.6% (n = 149) were men. The majority of the women (61.3%) were undergoing residency training at the time of their pregnancy. The announcement of the pregnancy to the supervisor(s) occurred on average in the 13th week of gestation (week 2–40). Before that, pregnant female physicians spent an average of 10 h per trimester (first trimester: 0–120 h; second trimester: 0–100 h) in the operating room. The main reason for women to continue surgical activity despite their (yet unreported) pregnancy was “own request”. 93% (n = 469) of the participants explicitly wished to be able to perform surgical activities in a safe setting during pregnancy. This response was independent of gender (p = 0.217), age (p = 0.083), specialty (p = 0.351), professional position (p = 0.619), and previous pregnancy (p = 0.142). In conclusion, there is an urgent need to offer female surgeons the possibility of continuing surgical activities during pregnancy. This handling would significantly increase the career opportunities for women who want to build up both a successful career and a family life.

## Highlights

• Globally a variety of laws regulate working and operating during pregnancy.
• In Austria, pregnant physicians must immediately disrupt surgical training as soon as their pregnancy is announced.
• The vast majority of Austrian physicians are in favor of enabling the performance surgical activities in a safe setting during pregnancy.
• This measure would significantly increase the career opportunities for women who want to build up both a successful career and a family life.

## 1 Introduction

Although the percentage of female physicians is steadily increasing worldwide, women remain significantly underrepresented in the higher echelon of hospital medicine, especially in surgical specialties [1,2]. In gynecology and obstetrics (OBGYN) the proportion of women is currently estimated to be 80% in Germany, and 55% in Austria [3]. The latter number, however, is much higher (approximately 80%), when female physicians in training are explicitly considered [4,5]. Given this demographic development, it is urgent to face current working regulations that negatively interfere with the educational training and career of female surgeons.

The topic of “operating during pregnancy” thus concerns a growing number of physicians. Due to the Maternity Protection Act from 1979 [1], the currently applicable legal regulation in Austria, performing surgeries during pregnancy is forbidden [6,7]. It states: “Expectant mothers may under no circumstances be employed in heavy physical work or in work or work procedures that are harmful to their organism or to the expectant child due to the nature of the work process or the working materials or equipment used.” This is further defined under §4 [1,7] as: “Work in which expectant mothers are exposed to the effects of substances hazardous to health, radiation hazardous to health, where harm cannot be ruled out” [8]. The Federal Ministry of Labor explains under the annotated Maternity Protection Act: “In the operating room, the employment of expectant and nursing mothers is inadmissible.” [9].

After the announcement of their pregnancy, female physicians are therefore prevented from continuing their surgical training. This handling of their situation leads to a prolongation of their residencies and is a considerable career disadvantage compared to their male colleagues, respectively. Hence, many female physicians disclose their pregnancy to their employers at a late stage to be able to maintain their surgical training for as long as possible. However, this puts the pregnant woman in a particularly challenging situation, as she cannot implement pregnancy-related safety precautions (e.g., not standing for long periods of time and not handling of infectious patients) and, thus, may endanger herself and her unborn child.

In Germany, a questionnaire was developed in 2010 within the framework of the „Career and Family” working group of the German Society for Gynecology and Obstetrics (DGGG) to determine the need for an amendment to the Maternity Protection Act. Two surveys based on this act were conducted and distributed among surgically active female physicians throughout Germany. The results of both questionnaires underlined the need for an amendment to the Maternity Protection Act and the ability for physicians to make the decision to perform surgery during pregnancy [3,6]. This subsequently led to a reform of the German Maternity Protection Act, which became effective on January 1, 2018 [10].

The study aimed to assess the current situation of how pregnant female surgeons handle their training under the actual restrictive legislature in Austria, especially in context of operative activity. Furthermore, we evaluated how parental leave and professional re-entry have been proceeded. Importantly, we aimed to identify needs for improvements to support women in building up both a surgical career and a family. Thus, a nationwide survey among surgically active physicians was conducted.

## 2 Materials and methods

### 2.1 Study design and participants

From June 1, 2021 to December 24, 2021 a survey was performed by the Austrian Society for Gynecology and Obstetrics (OEGGG) and the Young Forum of the OEGGG, a group representing all residents of OBGYN in Austria. 12 Austrian surgical societies were contacted, of which the following eight specialties agreed to participate in the survey: general surgery, orthopedic and trauma surgery, maxillofacial surgery, pediatric surgery, urology, otorhinolaryngology and dermatology. A web-based questionnaire entitled “Operating in pregnancy?” was e-mailed together with an invitation letter to all registered members of these societies. The participants were enrolled anonymously. After several reminders, 503 members in total responded to the survey. This corresponded to a response rate of 20%. To validate the questionnaire, a pre-test on 25 surgically active physicians from the above-mentioned disciplines was carried out prior to the main study. Following inconsistent responses and critical feedback, some of the questions were rephrased.

### 2.2 Questionnaire

The questionnaire included a total of 30 questions (18 questions for men and 28 questions for women) classified into the sub-areas of demographic variables, pregnancy and operative activity, parental leave, and professional re-entry. The response format of the questions varied; and included dichotomous questions, open-ended questions, questions to be answered according to a 3-point Likert scale (1 = "dissatisfied [−]"; 2 = "satisfied [+/−]"; 3 = "very satisfied [+]"), and classification questions. Both the survey design and data collection were carried out using the web-based survey tool SurveyMonkey.com (Momentive Europe UC, Dublin, Ireland).

### 2.3 Statistical evaluation

Statistical analysis was performed using IBM SPSS Statistics, Version 27 (IBM Corp., Armonk, NY, USA). Descriptive data were reported according to their scale level as a median with minimum and maximum or as a percentage (%) and absolute frequency (n). For group comparisons, the Mann-Whitney *U* test or the qi-square test was used. The question of whether pregnant women should be allowed to pursue surgical activities during pregnancy at their own request was evaluated with multivariate statistics (including the covariates of sex, age, specialty, professional position, and previous pregnancy), using binary logistic regression analysis. In all statistical procedures, two-sided testing was performed with a significance level of p < 0.05.

## 3 Results

### 3.1 Sample description

A total of 503 physicians participated in the survey, of which 73% were from the specialty of obstetrics and gynecology (n = 365), and 27% were from other surgical disciplines (n = 138), including general surgery, orthopedic and trauma surgery, maxillofacial surgery, pediatric surgery, urology, otorhinolaryngology and dermatology. A total of 70.4% (n = 354) of the respondents were women, and 29.6% (n = 149) were men. The baseline characteristics of the participants are shown in Table 1.

**Table 1:** Baseline characteristics of the entire study population (N = 503).

Parameter	number (N)	frequency (%)
***Age***		
20–25 years	2	0.4
26–30 years	60	11.9
31–35 years	106	21.1
36–40 years	108	21.5
41–45 years	48	9.5
>45 years	179	35.6
***marital status***		
Married	312	62.0
Living separately	11	2.2
Living together	135	26.8
Single	23	4.6
Single parent	4	0.8
Divorced	14	2.8
Widowed	4	0.8
***Current professional situation***		
Clinical Practical Year	5	1.0
Residency	159	31.6
Medical specialist	171	34
Senior physician	73	14.5
Senior physician in charge	38	7.6
Primary physician	57	11.3
***Selected specialty***		
Gynecology/obstetrics	365	72.6
Surgery	19	3.8
Orthopedics	3	0.6
Urology	84	16.7
Otorhinolaryngology	25	5.0
Opthalmology	1	0.2
Dermatology	4	0.8
Other	2	0.3
***Team size at current place of work***		
0–5	94	18.7
6–10	60	11.9
11–15	125	24.9
16–20	78	15.5
21–30	74	14.7
31–40	33	6.6
>40	39	7.7

### 3.2 Pregnancy and surgical activity

The majority of the female participants (n = 226, 63.8%) had one previous pregnancy, 130 (36.7%) had two pregnancies, and 33 (9.3%) had three or more pregnancies.

**Fig. 1:**
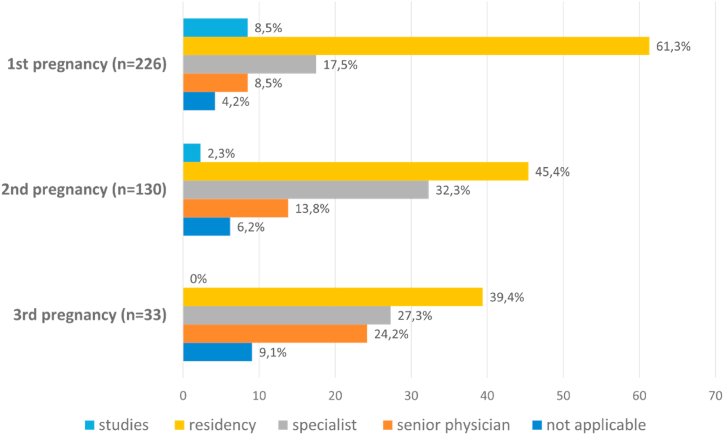
Career status at time of pregnancy.

At the time of pregnancy, most women were in residency training (Fig. 1).

**Fig. 2:**
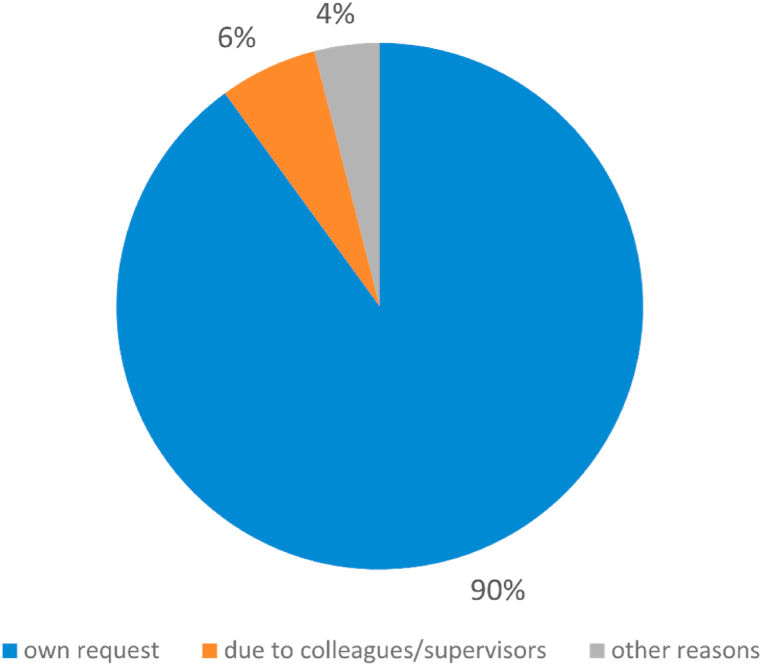
Reasons for pursuit of surgical activities during pregnancy.

On average, the announcement of the physicians’ pregnancy to their supervisor(s) occurred in the 13th week of gestation (week 2–40), and pregnant physicians left operative duties at the 15th week of gestation (week 2–38). In the first and second trimesters, pregnant female physicians spent an average of 10 h per trimester (first trimester: 0–120 h; second trimester: 0–100 h) in the operating room. In the third trimester, they spent an average of 5 h per trimester (0–60 h) in the operating room. The reasons why female physicians pursued surgical activities during pregnancy despite the applicable maternity protection law can be seen in Fig. 2.

**Fig. 3:**
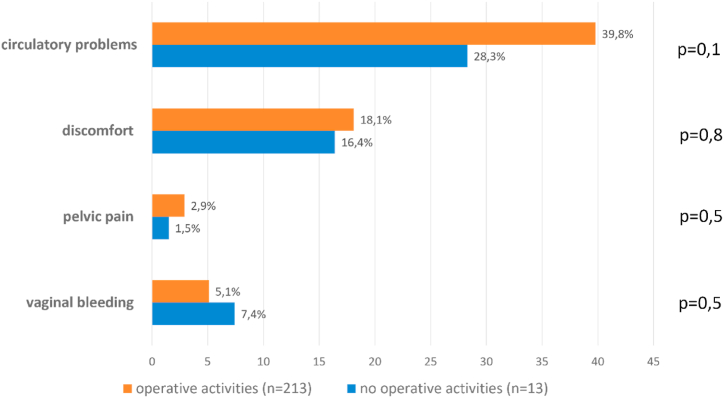
Pregnancy complaints in surgical active and non-active female physicians.

**Fig. 4:**
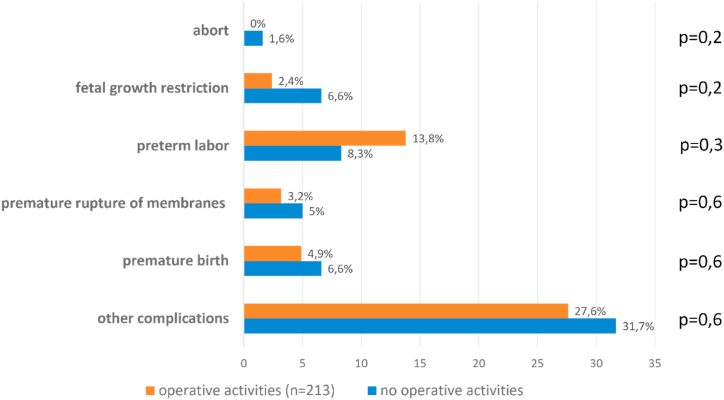
Pregnancy complications in surgical active and non-active female physicians.

Neither pregnancy complaints nor complications differed between surgically active (n = 213, 94.2%) and inactive female physicians (n = 13, 5.8%) (Fig. 3, Fig. 4).

The majority (n = 171, 80.3%) of female physicians who had been surgically active during one pregnancy indicated that they wanted to do so again in a subsequent pregnancy. Regarding satisfaction, 37.6% of the female physicians were dissatisfied with the training situation during pregnancy, 46.5% were satisfied, and 15.9% were very satisfied. When asked if pregnant women should be allowed to pursue surgical activities within a safe setting during pregnancy at their explicit request, 93.2% (n = 469) answered “Yes.” This was independent of gender (p = 0.217), age (p = 0.083), specialty (p = 0.351), professional position (p = 0.619), and previous pregnancy (p = 0.142).

### 3.3 Parental leave

Regarding the participants'current family situations, 63.8% (n = 226) of the women reported having at least one child, while this was true for 84.6% (n = 126) of men *(p < 0.0001).* The average number of children for both genders was two. Parental leave was taken by 89.4% (n = 202) of the women (for an average of 10 months per child; 3–36 months), but only by 17.5% (n = 22) of the men (for an average of two months per child; 0–7 months). In general, male participants (n = 149) were not motivated to take paternity leave because of “the supervisor(s)" (19.7%), their “career” (36.4%), or “other reasons” (e.g., “was not possible at the time”) (43.9%). For 94.5% of the male physicians, reasons such as “spending time with the children”, “fair sharing”, and „supporting the partner” were decisive factors for taking parental leave. Female physicians on parental leave continued their education in 55.1% of cases, while 33.1.% only engaged in a partial continuation of their education, and 11.8% did not continue their training during parental leave.

### 3.4 Professional re-entry

On average, 95.6% of the women (n = 216) returned to work after their pregnancies whereas 4.4% (n = 10) had not resumed their professional activities at the time of the survey.

After returning to work, 26.5% of the female physicians were unsatisfied with their training situation, 56.9% were satisfied, and 16.6% were very satisfied. For 22.1% of the female physicians, it was difficult to establish a routine in their operative work after re-entry, while this was only partly true for 36.6.% of the female physicians and was not true at all for 41.3% of the female physicians. A total of 8.5% of the female physicians reported that they had to re-learn operative techniques after a pregnancy-related break, while 33.9% reported that this was partially true, and 57.6% reported that this was not at all the case. The response rate for the survey was 20%.

## 4 Discussion

The majority of the participants in our study (93.2%) would support the possibility of performing surgical procedures during pregnancy. This is consistent with the findings of Knieper et al. and Fritze-Büttner et al. [3,6]. A worldwide comparison shows a great heterogeneity of legislation that regulate working and operating during pregnancy. While female surgeons in the United States work full time until the onset of labor, in Belgium, there is a ban on employment for at least one week antepartum and nine weeks postpartum. In Switzerland, like in Germany, pregnant physicians are allowed to continue their surgical activities during pregnancy, and maternity protection begins on the day of birth. Additionally, in Germany, there is a six-week ban on employment antepartum and an eight-week ban postpartum [[11], [12], [13]].

Austria has always been considered a showcase model. There is a general 8-week ban on employment before and an 8–12 week ban after delivery. Besides, there is a long period of paid maternity leave and the ability of both mothers and fathers to stay at home. Currently, however, female physicians in Austria are not allowed to continue surgical procedures from the time they announce their pregnancy [8,14]. Although the Maternity Protection Act is primarily aimed at protecting pregnant women and the fetuses, our study revealed that pregnancy complications did not differ between the two groups of surgically active and non-active female doctors. Indeed, there are studies suggesting otherwise, but they are mainly performed in the U.S. and therefore hardly comparable with Austria since they included a lot of female surgeons that had postponed their family planning, resulting in higher age at first pregnancy which is a risk factor per se for pregnancy complications [[15], [16], [17], [18]].

The proportion of women among medical students and practicing physicians worldwide has increased consistently over the past few decades [2,19], and more and more female patients are requesting care from female physicians [20]. Nevertheless, women continue to be underrepresented in surgical specialties compared to their male counterparts, especially in higher positions. A recently published systematic review by Hirayama et al. addressed, in addition to the delay of surgical training due to a pregnancy, further potential barriers of career advancement for female surgeons [1]. They identified two main problems: a rigid organizational structure designed for male surgeons and a persistent male dominance that gives male surgeons a sense of superiority over their female colleagues. Furthermore, a conflict in terms of work-life balance is still present among female surgeons, as they feel that they have to choose between a family and career under the current framework [[1], [21], [22], [23], [24], [25], [26], [27]. This is also consistent with our results, which show that 89.4% of the participating women took parental leave for an average of 10 months per child, while only 17.5% of the men took parental leave for an average of two months per child *(p < 0.0001).* Further challenges female surgeons have to face are a lack of female role models [[28], [29], [30], [31]] and gender discrimination [28,29,[32], [33], [34], [35], [36], [37]]. It is also often reported that female surgeons are forced to spend less time in the operating room due to administrative work and that their male colleagues are more involved in surgical training opportunities by leading consultants [38]. Consequently, surgical disciplines are those medical fields with the highest number of female physicians changing their specialty throughout their careers [19].

According to our needs assessment, there is a desire in Austria for a contemporary adaptation of the Maternity Protection Act, especially regarding the possibility of allowing physicians to continue their surgical activities during pregnancy. This could be the first step for Austrian female surgeons towards breaking the “glass ceiling” in surgical specialties. This fact has been stated by many studies around the world, meaning a disparity between the percentage in female surgeons in training and those who stay in the surgical field after residency or even obtain leadership or academic roles [[38], [39], [40], [41]].

In our view, adjustments should follow the reforms implemented in Germany a few years ago [3,6,10,42]. The changes to the German law came into force in 2018 and resulted in pregnant physicians being allowed to continue to work in the operating room and workplace conditions being outlined to help reduce risk to the pregnant doctors, including making a chair available, regulating how much standing activity is allowed, conducting mandatory hepatitis C and HIV screenings in patients before surgery, and wearing appropriate protective clothing [8,10,43].

These and other measures could also be implemented in Austria and would contribute to equal opportunities in the training of surgical residents. In view of the predicted shortage of doctors beginning in 2030, this reformation would enhance the attractiveness of surgical disciplines to women [44]. However, the exact legal regulations regarding the adapted working conditions, and the fact that this possibility is based on the willingness of the pregnant physicians, must be pre-supposed. Furthermore, it is imperative to ensure that pregnant physicians do not experience any disadvantages if they choose to not proceed with surgery during pregnancy. With these changes, it would no longer be necessary for pregnant physicians to report their pregnancies only at an advanced stage to avoid suffering a training disadvantage and, thus, to work longer under insecure working conditions. This existing circumstance is also clearly reflected in our survey, in which a large proportion of respondents stated that they had not left operative work until after the first trimester or had not reported their pregnancy to their employer until after the first trimester.

### 4.1 Strengths

This is the first need assessment among doctors in surgical specialties in Austria to address this question and assess the need for a change and a contemporary adaptation of the Maternity Protection Act. The results of our survey are clear and suggest that pregnant doctors should be able to decide by themselves if they want to pursue their surgical activities during pregnancy while complying with protective regulations. Also, we collected data on maternity and paternity leave, professional re-entry, possible pregnancy complications and the current practice among female surgeons which suggests that they often keep on their surgical activities without protective regulations due to the strict Maternal Protection Act in Austria.

### 4.2 Limitations

Although for online surveys a response rate of 20% is an acceptable value, low numbers of respondents in some subgroups (e.g., surgically non-active pregnant doctors) may contribute to a type II error. This should be kept in mind when interpretating data showing no differences between groups. Furthermore, a non-standardized and self-created questionnaire was used for the survey; however validation was done by a pre-test on 25 surgically active physicians. Finally, potential bias due to demographic values of the respondents could not be controlled within this study design; however, all characteristics have been presented and can be thus included in data interpretation.

## 5 Conclusion

In summary, our data clearly underline the urgent need to optimize the quality of training for pregnant physicians and to enable them to pursue surgical activities during pregnancy while complying with protective regulations. This reformation would significantly increase the career opportunities for women who want to build up both a successful career and a family life.

## Author contribution statement

Karin Windsperger: Conceived and designed the experiments; Performed the experiments; Analyzed and interpreted the data; Wrote the paper.

Nadja Taumberger and Philipp Foessleitner: Performed the experiments; Analyzed and interpreted the data; Wrote the paper.

Petra Pateisky: Conceived and designed the experiments; contributed reagents, materials, analysis tools or data.

Taja Bracic: Analyzed and interpreted the data.

Bettina Toth: Analyzed and interpreted the data; Contributed reagents, materials, analysis tools or data.

## Data availability statement

Data will be made available on request.

## Declaration of competing interest

The authors declare that they have no known competing financial interests or personal relationships that could have appeared to influence the work reported in this paper.
